# Global outcomes, surgical teams and COVID-19 pandemic: Will the same objectives of global surgery persist?

**DOI:** 10.1016/j.amsu.2021.103002

**Published:** 2021-10-30

**Authors:** Jorge Alberto Nuñez-Gamez, Paola Andrea Medina-Bravo, Nicolás Felipe Piñeros-López, Gersson A. Contreras, Mary Elena Rosero-Burgos, Ivan David Lozada-Martínez, Sabrina Rahman

**Affiliations:** School of Medicine, Universidad del Sinú, Tv. 54 #41-117, Cartagena, Colombia; School of Medicine, Universidad El Bosque, Ak. 9 #131a-20, Bogotá, Colombia; School of Medicine, Universidad del Rosario, Calle 12C Nº 6-25, Bogotá, Colombia; School of Medicine, Universidad de Santander, Calle 70 N° 55-210, Bucaramanga, Colombia; School of Medicine, Fundación Universitaria San Martín, Cl 18 #42a-44, Pasto, Colombia; Medical and Surgical Research Center, Future Surgeons Chapter, Colombian Surgery Association, Bogotá, Colombia; Department of Public Health, Independent University- Bangladesh, Dhaka, Bangladesh

Dear Editor,

The sudden appearance of the COVID-19 pandemic in the world, generated changes with respect to the global objectives of several disciplines of biomedical sciences, thanks to the redistribution of investments, modification in the development of academic and assistance activities, specialized attention, among other causes; which caused that the general objectives of each area of knowledge were quickly revised and validated. In surgery, in the last decade, objectives were set for the year 2030 taking into account the substantial progress that this discipline has made in the 21st century [1]. Access to essential and emergency surgical services and the global reduction of the burden of disease is the main objective of academic and global surgery [1]. Funk et al. [2] determined that approximately 2 billion people do not have access to these services, being more intense in low- and middle-income countries [2]. The promotion of academic surgery [3], improvement in the training of residents [4], the design of modes and strategies that improve the final and functional outcomes in the care practice [5], highly specialized training, student participation and training in the surgical field [6,7], professionalism in surgery [8], gender equity [9], among others [5]; constitute the rest of the proposed objectives, in order to make surgery an evidence-based discipline that guarantees results, optimizes the patient's experience, improves the doctor-patient relationship and facilitates the development of the practice with the best cost-effectiveness and cost-utility [1,[3], [4], [5]]. However, after the emergence of the pandemic, new problems came to light and it became evident that surgical progress has been slow in some parts of the world [3,6,8]. Therefore, it is valid to ask, will the same global surgical goals persist?

Luan et al. [10] recently criticized long-term outcome findings and global surgery research, stating that little is known with certainty about the impact of certain interventions on surgical disease burden [10]. For the solution of this problem, they propose some points that can represent new objectives of global surgery: enhancing systems to facilitate long-term follow up and care, expanding availability and adoption of electronic medical record systems, and collaboration with local surgeons in the development of international cross-organizational registries and standardized quality measures [10], clear and reproducible objectives. Surgical teams played a fundamental role in the control of the COVID-19 pandemic, mainly through the assistance of these teams in other departments such as critical care or emergency, facilitating the performance of minimally invasive interventions for the support of critically ill patients [11]. However, the emergence of surgical interest groups as strategies for the transition of medical students, residents and teachers to the virtuality was fundamental [6,7,12]. These groups contribute substantially to training, preparation on professionalism issues, resolution of problem questions with evidence-based theoretical answers, assistance support, mentoring, among others; therefore, they have positioned themselves as another point to highlight and reinforce in global surgery [6,7,12]. This goes hand in hand with the improvement of academic surgery by professors and trainers, who have stated that there are 7 objectives that an academic surgeon should pursue, among which the following stand out: identifies complex clinical problems ignored or thought unsolvable by others, observes outcomes to further improve and innovate, and trains the next generation of surgeons and scientists [5].

The creation of centers specialized in surgical attention and care by sub-specialties, and the investment in purchasing and training in robotic surgery and high complexity technological equipment, are a complex but necessary topic to continue discussing [13,14]. It will be very difficult to cope with future global public health crises such as new pandemics, if we do not have the necessary tools to optimize surgical care and facilitate patient flow, hospital stay and control the risk of postoperative complications, morbidity and mortality [14]. Hence, it is important to constantly evaluate the progress of the goals set. The evidence suggests that there is much room for improvement and that low- and middle-income countries still need to strengthen their health policies on health care [15]. Several international prospective multicenter studies are currently underway to determine the quality of life and outcomes of surgical care for high-impact conditions such as cancer management [16], chronic noncommunicable diseases [17], risk and postoperative care [18], therefore, there is still much to be known and researched in surgery. In this order of ideas, it can be said that taking into account that there is much to be achieved with respect to the goals previously set by global surgery, new objectives arise as the evidence is discussed critically and constructively, and together with COVID-19, new challenges have become evident to which practical and quality solutions have been proposed, with results that tend to be favorable. Academic surgery, professionalism in surgery and the participation of medical students in global surgery are innovative points that are likely to have good results in the year 2030.

## Data statement

Data sharing is not applicable to this article as no new data were created or analysed in this study.

## Ethical approval

Not applicable.

## Sources of funding

None.

## Author contribution

All authors equally contributed to the analysis and writing of the manuscript.

## Research registration Unique Identifying number (UIN)

1. Name of the registry: Not applicable.
2. Unique Identifying number or registration ID: Not applicable.
3. Hyperlink to your specific registration (must be publicly accessible and will be checked): Not applicable.

## Guarantor

Sabrina Rahman, Department of Public Health, Independent University- Bangladesh, Dhaka, Bangladesh. E-mail: sabrinaemz25@gmail.com.

## Provenance and peer review

Commentary, internally reviewed.

## Declaration of competing interest

All authors declare that there exist no commercial or financial relationships that could, in any way, lead to a potential conflict of interest.
